# Direct and Indirect Effects of Five Factor Personality and Gender on Depressive Symptoms Mediated by Perceived Stress

**DOI:** 10.1371/journal.pone.0154140

**Published:** 2016-04-27

**Authors:** Song E. Kim, Han-Na Kim, Juhee Cho, Min-Jung Kwon, Yoosoo Chang, Seungho Ryu, Hocheol Shin, Hyung-Lae Kim

**Affiliations:** 1 Department of Biochemistry, School of Medicine, Ewha Womans University, Seoul, South Korea; 2 Center for Cohort Studies, Total Healthcare Center, Kangbuk Samsung Hospital, School of Medicine, Sungkyunkwan University, Seoul, South Korea; 3 Department of Health Sciences and Technology, SAHIST, Sungkyunkwan University, Seoul, South Korea; 4 Biostatistics and Clinical Epidemiology Center, Research Institute for Future Medicine, Samsung Medical Center, School of Medicine, Sungkyunkwan University, Seoul, South Korea; 5 Department of Health, Behavior and Society and Epidemiology, Johns Hopkins Bloomberg School of Public Health, Baltimore, Maryland, United States of America; 6 Department of Laboratory Medicine, Kangbuk Samsung Hospital, Sungkyunkwan University, School of Medicine, Seoul, Republic of Korea; 7 Department of Occupational Medicine, Kangbuk Samsung Hospital, School of Medicine, Sungkyunkwan University, Seoul, South Korea; 8 Department of Family Medicine and Health Screening Center, Kangbuk Samsung Hospital, School of Medicine, Sungkyunkwan University, Seoul, South Korea; University of Vienna, AUSTRIA

## Abstract

This study was designed to investigate associations among five factor personality traits, perceived stress, and depressive symptoms and to examine the roles of personality and perceived stress in the relationship between gender and depressive symptoms. The participants (N = 3,950) were part of a cohort study for health screening and examination at the Kangbuk Samsung Hospital. Personality was measured with the Revised NEO Personality Inventory (NEO-PI-R). Depressive symptoms were assessed using the Center for Epidemiologic Studies Depression Scale (CES-D). Perceived stress level was evaluated with a self-reported stress questionnaire developed for the Korea National Health and Nutrition Examination Survey. A higher degree of neuroticism and lower degrees of extraversion, agreeableness, and conscientiousness were significantly associated with greater perceived stress and depressive symptoms. Neuroticism and extraversion had significant direct and indirect effects (via stress as a mediator) on depressive symptoms in both genders. Agreeableness and conscientiousness had indirect effects on depression symptoms in both genders. Multiple mediation models were used to examine the mediational roles of each personality factor and perceived stress in the link between gender and depressive symptoms. Four of the personality factors (except openness) were significant mediators, along with stress, on the relationship between gender and depressive symptoms. Our findings suggest that the links between personality factors and depressive symptoms are mediated by perceived stress. As such, personality is an important factor to consider when examining the link between gender and depression.

## Introduction

Personality traits describe individual characteristics such as cognitive, emotional, and behavioral aspects that may play a role in diatheses or an increased propensity to psychopathologic states [1, 2] including depressive symptoms. The Five Factor Model (FFM) of personality defines the five dimensions of personality as neuroticism, extraversion, openness to experience, agreeableness, and conscientiousness. Neuroticism is associated with negative emotions such as anxiety, fear, and anger [3, 4]. Extraversion is the tendency to be active and sociable [5]. Openness to experience is the tendency toward preferring unconventional ideas and experiencing diverse emotions [6]. Agreeableness refers to interpersonal characteristics such as altruistic and cooperative tendencies. Conscientiousness is characterized by persistence, organization, and goal-directed behavior [7]. Previous studies have shown that personality characteristics such as higher neuroticism and lower extraversion, conscientiousness, and agreeableness were associated with the onset and prognosis of depressive disorders [8, 9].

Different types of personality may be associated with variable reactivity to stress, such as emotional regulation or coping styles [1, 10]. Higher neuroticism has been linked with increased negative feelings and maladaptive behavioral responses to stressful experiences [4]. Extraverted persons are likely to experience more positive affect and less stress [11]. Agreeable individuals are likely to avoid interpersonal conflict and experience less social stress [12]. Higher conscientiousness is associated with more effective coping strategies such as active problem solving to deal with stress [11]. Thus, individual differences in reaction and perception of stress after experiencing negative life events may depend on their personality traits.

It is well known that the majority of depressive episodes are preceded by stressful life events, and severe stressful experiences increase the risk of developing depression [13]. Perceived levels of stress after negative life events differ across individuals [14], and specific factors may contribute to differences in vulnerability to stress, which in turn, increases the likelihood of developing depressive symptoms [15]. Among various stress vulnerability factors, a high neuroticism level is longitudinally associated with episodic stress and depressive episodes [16]. Higher neuroticism and lower extraversion partially account for depressive or social anxiety, and these personality traits explain shared associations between life stress and mood disorders [17]. A previous study examining the relationship between five factor personality traits and negative mood reported that perceived stress mediates these relationships [18]. Conscientiousness is known to be associated with stress management and tolerance [18] and lower risk of depression, but the links between agreeableness and openness with stress or depression are inconsistent [4, 8, 11]. Although many studies have reported relationships between two of the three variables, there is still limited understanding regarding the interrelationships among personality, stress, and depressive symptoms.

Depression is about twice as common in women, and greater depressive symptoms are consistently found in women compared to men across Western and Asian countries [18] including South Korea [19]. A diathesis stress model has been proposed to explain gender-related vulnerability to depression [20]. Women are more likely than men to experience episodic stress and emotional distress in daily events [21], which partially mediates gender differences in depression [22]. Personality traits have also been suggested as a possible mechanism of gender difference in depression [23]. Specifically, neuroticism tends to be higher in women than men [24]. A high degree of neuroticism was prospectively associated with later experiences of negative life events in adolescent girls and served as vulnerability factor of depression [25]. It was previously reported that neuroticism was a strong mediator of the association between gender and depression in addition to various social and psychological factors [26]. However, the effects of other FFM personality dimensions on the hypothesized links among stress, gender, and depressive symptoms remain unclear.

We hypothesized that personality traits would affect depressive symptoms by acting as vulnerability factors of perceived stress after experiencing negative events and that the effects of personality on perceived stress might mediate the association between gender and depressive symptoms. We tested two specific aims in the present study. First, we examined associations of each of the five personality factors and depressive symptoms through perceived stress. Secondly, we examined the mediating roles of each personality factor and perceived stress in the relationship between gender and depressive symptoms.

## Materials and Methods

### Participants

The participants in this study came from a cohort that underwent health screening and examination at the Kangbuk Samsung Hospital in Seoul, South Korea. All subjects provided information about socio-demographic characteristics, dietary and health-related behaviors, and family and medical history of a wide range of diseases. Among the 4225 participants aged 19–69, individuals with a history of psychiatric problems (N = 183) or cancer (N = 97) were excluded in the current study. Participants completing all measures of personality, stress, and depression were included, for a total of 3,950 participants (men, N = 1,134; women N = 2,816) were finally included. The cohort’s characteristics were previously described in detail [27].

### Statement of Ethics

This study was approved by the Institutional Review Board of Ewha Womans University Mokdong Hospital. We obtained written informed consent from all participants. All applicable institutional and governmental regulations concerning the ethical use of human volunteers were followed during the research.

### Measures

Depressive symptoms were assessed using the Center for Epidemiologic Studies Depression Scale (CES-D), which consists of self-reported responses to a 20-item questionnaire. The CES-D measures current depressive symptoms during the past week with a 4-point Likert scale. The total depressive score is the summation of all scores and ranges from 0 to 60. Stress level was evaluated by a self-reported stress questionnaire developed for the Korea National Health and Nutrition Examination Survey [28]. The questionnaire for perceived stress consisted of one item inquiring about the categories of stressful life events that had been experienced during the past month and nine items inquiring about stress levels due to those events. Stress levels were rated on a 5-point Likert scale (0: never; 4: very often). Total stress scores ranged from 0 to 36, which was the summed score of all items. The Cronbach’s alpha coefficients for CES-D and stress questionnaires were 0.996 and 0.980, respectively. Missing responses for CES-D (3.91%) and stress questionnaires (3.46%) were imputed with multiple imputation methods using “mice” library in R [29]. We used the predictor variables including demographic factors such as age, marriage, education, and employment status as candidate risk factors of depression according to the previous literature [30]. We also added neuroticism as a predictor variable to impute for CES-D and stress score because neuroticism has strong associations with these variables.

The Revised NEO Personality Inventory (NEO-PI-R) was used to measure each of the FFM personality domains (neuroticism, extraversion, openness to experience, agreeableness, and conscientiousness). The Korean version of the Revised NEO Personality Inventory showed good reliability and validity [31]. A robust factor structure has been replicated with this instrument in various cultures including Korea [32]. The instrument is comprised of items about personal characteristics rated on a 5-point Likert scale. Each of the personality factors consists of the six facets that define each factor. The neuroticism factor includes anxiety, anger, depression, self-consciousness, impulsiveness, and vulnerability. The extraversion factor includes warmth, gregariousness, assertiveness, activity, excitement-seeking, and positive emotions. Openness to experience includes fantasy, aesthetic feelings, actions, ideas, and values. Agreeableness includes trust, straightforwardness, altruism, compliance, modesty, and tender-mindedness. Conscientiousness includes competence, order, dutifulness, achievement-striving, self-discipline, and deliberation. The Cronbach’s alpha coefficients for neuroticism, extraversion, openness, agreeableness, and conscientiousness were 0.908, 0.905, 0.809, 0.820, and 0.906, respectively. Raw scores were converted to T-scores (mean = 50, standard deviation = 10) using Korean combined-sex norms (N = 7418).

### Statistical Analysis

Pearson’s chi-square tests and independent sample t-tests were conducted to examine gender differences in demographic characteristics, stress, depression, and personality scores. Cohen’s d was calculated as an effect size for t-tests. Pearson correlation analysis was performed to determine the association among the study variables. SAS 9.3 was used for all statistical analyses.

The mediation effect is referred to as the indirect effect of the independent variable (IV) on the dependent variable (DV) through the intervening variables. The mediation effect occurs when the correlation between the IV and DV is eliminated (complete mediation) or reduced (partial mediation) when the mediator is controlled for. To examine the effect of personality on depressive symptoms mediated by stress (mediation model 1), mediation analysis was conducted using an SAS macro provide by Preacher and Hayes (2008) [33]. Using mediation analysis, the direct effects of the IV and the indirect effect through mediator (M) on the DV were estimated. The indirect effect of the IV on DV via M was quantified as a×b. Path c represents the total effect of each personality factor on depressive symptoms, and path c’ is the direct effect of personality factor on depressive symptoms. The total effect (c) of IV on DV equals the sum of the direct and indirect effects (*c* = *c’*+*ab*) [33].

**Fig 1:**
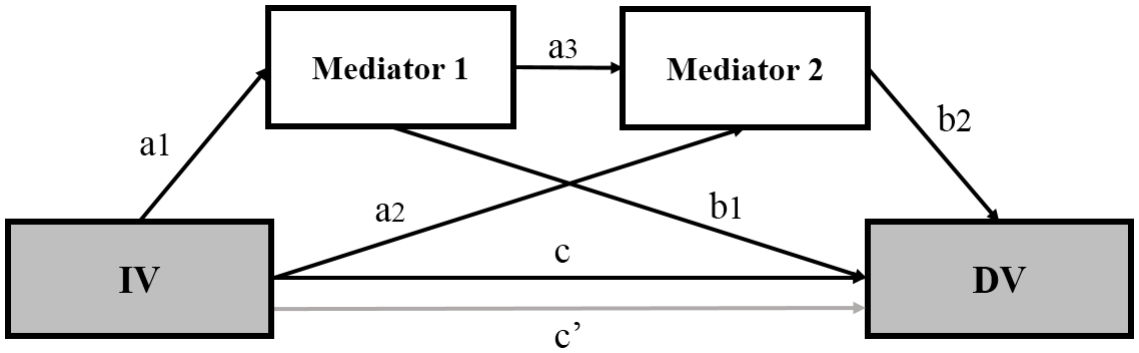
Multiple mediation model. The relationships between gender (IV, independent variable) and depressive symptoms (DV, dependent variable) mediated by each personality factor (M1, mediator 1) and stress (M2, mediator 2). Path a1 represents the effect of IV on M1, and path a2 represents the effect of IV on M2. Path a3 is the effect of M1 on M2. Path b1 and b2 represent M1 and M2 on DV, respectively. Path c represents the total effect of IV on DV, and path c’ is the direct effect of IV on DV.

We also tested multiple serial mediation effects of personality and stress on the association between gender and depression [34] (mediation model 2). For the multiple mediation analysis, three indirect effects of the IV on DV through M1 and M2 (IV→M1→Y, IV→M2→Y, IV→M1→M2→DV) can be estimated (Fig 1). Path a1 represents the effect of gender on the personality, and path a2 represents the effect of gender on the stress. Path a3 is the effect of personality on stress. Path b1 and b2 represent the effect of personality and stress on depression, respectively. The total effect (c path) of gender on depressive symptoms was quantified as sum of the direct and indirect effects of gender on depressive symptoms. Path c’ is the direct effect of gender on depressive symptoms. The indirect effect of the IV on DV via M1 was quantified as a1×b1, and the indirect effect of IV on DV via M2 was quantified as a2×b2. The indirect effect via M1 and M2 equals subtraction of direct effect (c') and indirect effects via M1 (a1×b1) and M2 (a2×b2) from total effect (c) (c—[c’+ (a1×b1) + (a2×b2)]).

To test the mediation effect, 5000 bootstrapped resamples and a 95% confidence interval were applied to construct the indirect path. Bias-corrected confidence intervals that did not include 0 were considered significant for the indirect effect of the IV on DV via a mediator. Effect size in mediation analysis was assessed with completely standardized indirect effect size (Effect size_cs_) indicating that DV is expected to decrease by the magnitude of effect size standard deviations for every one standard deviation increase in IV indirectly via mediators. Effect size_cs_ is interpreted as small (0.01), medium (0.09), and large (0.25) [35, 36]. Socio-demographic factors, which are known to be correlated with depression [37, 38], such as age, education (less than high school, college), working status (employed, not employed), marital status (single, married/cohabiting) were included as covariates in all mediation models to control for their potential confounding effects on depressive symptoms. The mediation analyses without covariates were also conducted in order to validate our proposed mediation models.

## Results

### Descriptive Analysis and Gender Differences among Study Variables

Descriptive statistics of demographic and psychological variables by gender are presented in Table 1. The mean stress level was significantly higher in women compared to men (t = -9.04, *df* = 2480.16, p<0.001). Mean depression scores measured by the CES-D were higher in women than in men (t = -12.26, *df* = 2809.56, p<0.001). Scores for neuroticism (t = -17.37 = 2260.17, p<0.001), openness to experience (t = -11.33, *df* = 2652.62, p<0.001), and agreeableness (t = -4.20, *df* = 2231.34, p<0.001) were higher in women than men, while extraversion (t = 2.01, *df* = 2205.86, p = 0.045) and conscientiousness (t = 12.77, *df* = 3948, p<0.001) was lower in women than men.

**Table 1:** Demographic characteristics.

	Men		Women			
	Mean^a^ or Frequency^b^	Range	Mean^a^ or Frequency^b^	Range	p-value	Cohen’s d
**Age**^a^	39.86 (8.8)	20–69	34.63 (6.16)	19–69	<0.001	
**Marital status**						
Marriage/cohabitation^b^	853 (75.2)		2209 (78.4)		0.032	
**Education**						
≥College^b^	1049 (92.5)		2435 (86.5)		<0.001	
**Working status**						
Employed^b^	1001 (88.3)		1631 (57.9)		<0.001	
**Stress**^a^	16.01 (5.92)	9–45	18.01 (7.08)	9–42	<0.001	-0.31
**Depression (CES-D)**^a^	9.79 (5.09)	0–48	12.24 (6.90)	0–34	<0.001	-0.40
**Personality**						
N^a^	51.43 (8.42)		56.70 (9.15)		<0.001	-0.60
E^a^	43.90 (9.26)		43.23 (9.08)		0.045	0.07
O^a^	47.07 (9.38)		51.13 (11.99)		<0.001	-0.38
A^a^	45.92 (10.30)		47.47 (11.04)		<0.001	-0.15
C^a^	46.06 (9.02)		41.95 (9.20)		<0.001	0.45

*Note*. N, neuroticism; E, extraversion; O, openness to experience; A, agreeableness; C, conscientiousness

^a^ Quantitative variable; mean (SD), p-value and Cohen’s d from t-test are shown.

^b^ Nominal scale; frequency (%) and p-value from χ^2^ test are shown.

### Correlation Analysis

The bivariate correlation analysis results among personality, stress, and depressive symptoms in all subjects are shown in Table 2. Higher perceived stress levels correlated with a higher degree of neuroticism (men, r = 0.464, p<0.01; women, r = 0.472, p<0.01) and lower degrees of extraversion (men, r = -0.225, p<0.01; women, r = -0.213, p<0.01), agreeableness (men, r = -0.157, p<0.01; women, r = -0.170, p<0.01), and conscientiousness (men, r = -0.160, p<0.01; women, r = -0.163 p<0.01). Openness to experience was correlated with stress level only in men, but the association was small (r = -0.06, p<0.05). Higher depression symptoms were significantly related to greater scores in neuroticism (men, r = 0.358, p<0.01; women, r = 0.393, p<0.01) and lower scores in extraversion (men, r = 0.242, p<0.01; women, r = 0.197, p<0.01), agreeableness (men, r = -0.121, p<0.01; women, r = -0.107, p<0.01), and conscientiousness (men, r = -0.195, p<0.01; women, r = -0.123, p<0.05).

**Table 2:** Bivariate correlations among personality, stress, and depression.

**Total (N = 3950)**			**1**	**2**	**3**	**4**	**5**	**6**	**7**
	**1**	**N**							
	**2**	**E**	-0.315**						
	**3**	**O**	0.009	0.392**					
	**4**	**A**	-0.227**	0.087**	0.029				
	**5**	**C**	-0.493**	0.307**	0.089**	0.152**			
	**6**	**Stress**	0.484**	-0.218**	0.024	-0.157**	-0.197**		
	**7**	**Depression**	0.409**	-0.208**	0.051**	-0.097**	-0.167**	0.652**	
**Men (N = 1134)**			**1**	**2**	**3**	**4**	**5**	**6**	**7**
	**1**	**N**							
	**2**	**E**	-0.441**						
	**3**	**O**	-0.153**	0.453**					
	**4**	**A**	-0.306**	0.159**	0.049				
	**5**	**C**	-0.540**	0.422**	0.132**	0.295**			
	**6**	**Stress**	0.464**	-0.225**	-0.060*	-0.157**	-0.218**		
	**7**	**Depression**	0.358**	-0.242**	-0.033	-0.121**	-0.195**	0.560*	
**Women (N = 2816)**			**1**	**2**	**3**	**4**	**5**	**6**	**7**
	**1**	**N**							
	**2**	**E**	-0.275**						
	**3**	**O**	0.000	0.389**					
	**4**	**A**	-0.234**	0.064**	0.009				
	**5**	**C**	-0.440**	0.265**	0.123**	0.123**			
	**6**	**Stress**	0.472**	-0.213**	0.020	-0.170**	-0.163**		
	**7**	**Depression**	0.393**	-0.197**	0.037*	-0.107**	-0.123*	0.666**	

*Note*. N, neuroticism; E, extraversion; O, openness to experience; A, agreeableness; C, conscientiousness

* *p*<0.05,

** *p*<0.01

### Direct and Indirect Effects of each Personality Factor on Depression via Stress

The first mediation model (model 1) tested how the personality factors were related with depressive symptoms via their effects on perceived stress (Table 3). The mediation analysis was performed separately for each gender because of significant gender differences in personality traits, stress, and depressive symptoms. Neuroticism affected depression directly (c’, men, coefficient: 0.077, SE = 0.016, p<0.001; women, coefficient: 0.077, SE = 0.012, p<0.001), as well as indirectly through stress (a×b, men, coefficient: 0.139, 95% CI: 0.113, 0.166; women, coefficient: 0.216, 95% CI: 0.194, 0.241) in both genders. The direct and indirect paths of extraversion to depression were also significant in both genders (c’, men, coefficient: -0.071, SE = 0.014, p<0.001; women, coefficient: -0.039, SE = 0.010, p<0.001; a×b, men, coefficient: -0.067, 95% CI: -0.087, -0.050; women, coefficient = -0.103, 95% CI: -0.120, -0.085). There was a significance in the indirect path, not in the direct path, between agreeableness and depression via stress (a×b, men, coefficient: -0.039, 95% CI: -0.058, -0.022; women, coefficient: -0.061, 95% CI: -0.078, -0.045) because the total effect (c path) of agreeableness on depression became non-significant after controlling for stress (c’ path). Conscientiousness was indirectly linked to depression via stress in women and men (a×b, men, coefficient: -0.064, 95% CI: -0.085, -0.045; women, coefficient: -0.073, 95% CI: -0.093, -0.051). The direct effect of conscientious on depression was only significant in men (c’, coefficient: -0.035, SE = 0.014, p<0.05). Openness to experience had neither a direct (c’, coefficient: 0.009, SE = 0.008, p = 0.27) nor indirect effect (a×b, coefficient: -0.004, 95% CI: -0.018, 0.011) on depression in women, but the indirect effect was significant in men (a×b, coefficient: -0.021 95% CI: -0.042, -0.002). Effect sizes_cs_ for the significant mediation model were 0.03–0.23 for men and 0.09–0.028 for women, which are suggested to be a small to medium effect size for men, and moderate to large effect size for women (S1 Table). The mediation models without covariates showed similar findings (S2 Table).

**Table 3:** Mediation effects of stress in the associations between personality and depression.

		Effect of IV on M		Effect of M on DV		Total effect		Direct effect		Indirect effect		
	IV	a^#^	SE	b^##^	SE	c^$^	SE	c'^$ $^	SE	a×b^&^	CI lower	CI upper
Men	N	0.324***	0.019	0.428***	0.023	0.216***	0.017	0.077***	0.016	0.139^†^	0.113	0.166
	E	-0.149***	0.019	0.453***	0.021	-0.139***	0.016	-0.071***	0.014	-0.067^†^	-0.088	-0.050
	O	-0.043*	0.019	0.477***	0.021	-0.036*	0.016	-0.016	0.013	-0.021^†^	-0.042	-0.002
	A	-0.082***	0.018	0.477***	0.021	-0.047**	0.015	-0.008	0.013	-0.039^†^	-0.058	-0.022
	C	-0.137***	0.020	0.468***	0.021	-0.099***	0.017	-0.035*	0.014	-0.064^†^	-0.085	-0.045
Women	N	0.360***	0.013	0.599***	0.016	0.293***	0.013	0.077***	0.012	0.216^†^	0.194	0.239
	E	-0.162***	0.013	0.633***	0.014	-0.142***	0.013	-0.039***	0.010	-0.103^†^	-0.120	-0.085
	O	-0.006	0.011	0.645***	0.014	0.005	0.011	0.009	0.008	-0.004	-0.018	0.011
	A	-0.095***	0.012	0.647***	0.014	-0.054***	0.012	0.007	0.009	-0.061^†^	-0.078	-0.045
	C	-0.113***	0.015	0.643***	0.014	-0.083***	0.014	-0.010	0.011	-0.073^†^	-0.093	-0.051

*Note*. N, neuroticism; E, extraversion; O, openness to experience; A, agreeableness; C, conscientiousness; IV, independent variable; DV, dependent variable; M, mediator; SE, standard error; CI, confidence interval

^#^ a, the effect of each personality factor on stress

^##^ b the effect of stress on depressive symptoms

^$^ c, the total effect of each personality factor on depressive symptoms

^$ $^ c’, the direct effect of each personality factor on depressive symptoms

^&^ a×b, the indirect effect of each personality factor on depressive symptoms via stress

* *p*<0.05,

**p<0.01,

***p<0.001

^†^ significant indirect effect

### Direct and Indirect Effects of Gender on Depression via each Personality Factor and Stress

**Fig 2:**
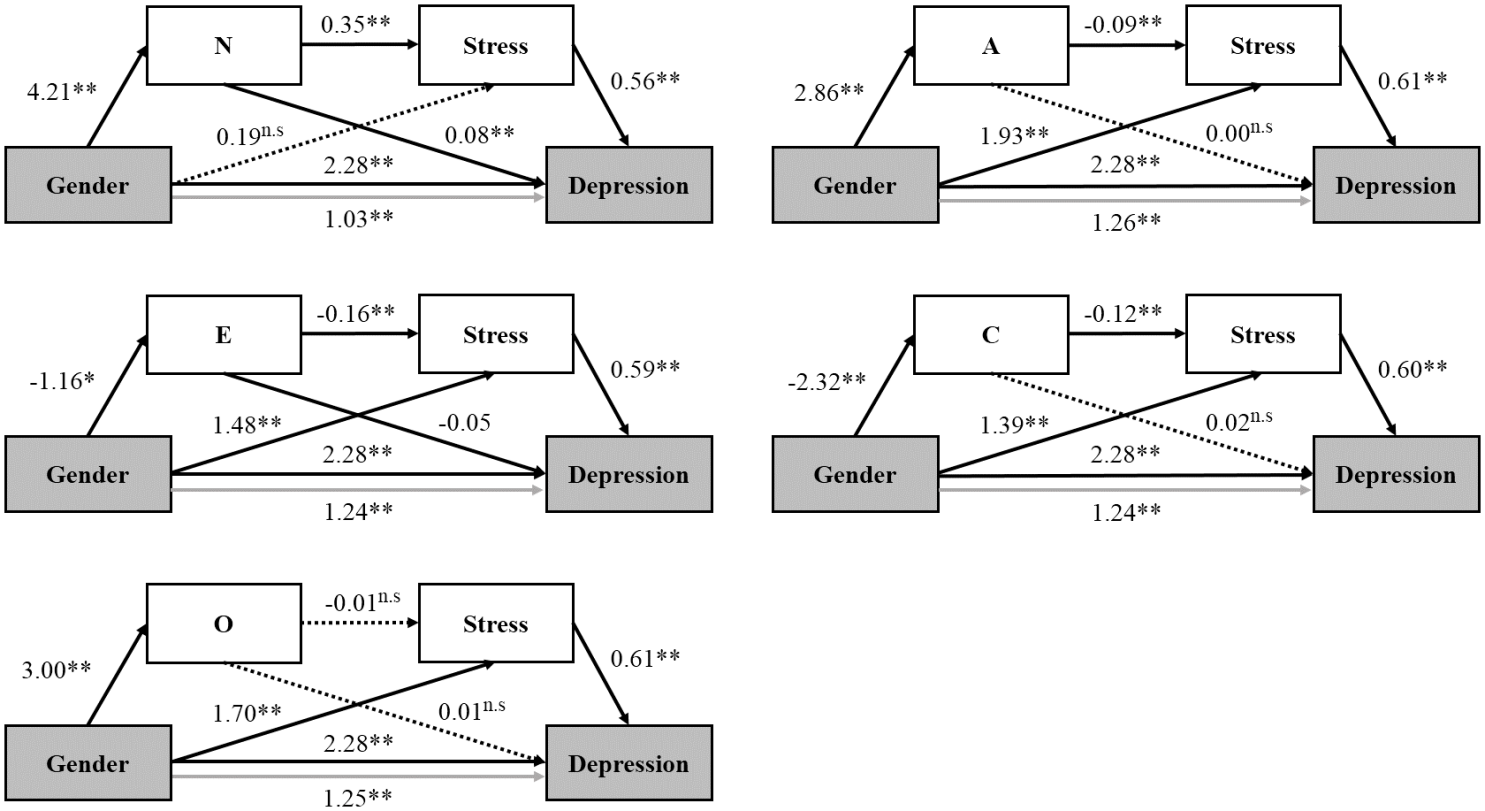
The association between gender and depressive symptoms with each pathway in the multiple mediation model. The pathways represented by arrows correspond to Fig 1. Each arrow with a solid line represents the significant path between variables, and the arrow with a dashed line represents the non-significant path. The path estimation from the mediation analysis shown with each arrow is the unstandardized coefficient. *Note*. Gender was coded 0 = man, 1 = women; N, neuroticism; E, extraversion; O, openness to experience; A, agreeableness; C, conscientiousness; n.s., non-significant; *p<0.01, **p<0.001.

The next analysis was designed to examine the direct effect of gender (IV) on depression (DV), and the indirect effects when mediated by personality (M1) and stress (M2) were significant (Fig 1). Fig 2 depicts each pathway between gender and depressive symptoms with multiple mediators in the second mediation model (model 2). The effects of gender on the five personality factors were significant, indicating that female gender was associated with higher neuroticism (coefficient: 4.207, SE = 0.35, p<0.001), openness (coefficient: 2.995, SE = 0.44, p<0.001) and agreeableness (coefficient: 2.857, SE = 0.42, p<0.001) but lower extraversion (coefficient: -1.155, SE = 0.38, p<0.01) and conscientiousness (coefficient: -2.316, SE = 0.35, p<0.001).

Higher neuroticism (coefficient: 0.351, SE = 0.01, p<0.001), and lower extraversion (coefficient: -0.158, SE = 0.01, p<0.001), agreeableness (coefficient: -0.092, SE = 0.01, p<0.001) and conscientiousness (coefficient: -0.120, SE = 0.01, p<0.001) were associated with higher perceived stress. Openness was related with neither perceive stress (coefficient: -0.01, SE = 0.01, p = 0.19) nor depressive symptoms (coefficient: 0.01, SE = 0.01, p = 0.28). There was a significant direct effect of gender on depression after controlling for personality and stress.

The mediational effects of the five personality factors and perceived stress on the association between gender and depressive symptoms are shown in Table 4. The indirect effect of gender on depression through the pathway via neuroticism and stress was significant (M1&M2, coefficient: 0.830, 95% CI: 0.682, 0.994). Gender exerted a significant indirect effect on depression via neuroticism (a1×b1, coefficient: 0.314, 95% CI: 0.224, 0.417) but not via stress after controlling for neuroticism (a2×b2, coefficient: 0.105, 95% CI: -0.144, 0.348).

**Table 4:** Multiple mediation effects through personality and stress in the association between gender and depression.

	Indirect effect via M1 (a1×b1^#^)			Indirect effect via M2 (a2×b2^$^)			Indirect effect via M1 & M2^&^			Indirect effect (total)		
	Coefficient	CI lower	CI upper	Coefficient	CI lower	CI upper	Coefficient	CI lower	CI upper	Coefficient	CI lower	CI upper
N	0.314	0.224	0.418	0.105	-0.144	0.348	0.830	0.682	0.994	1.248	0.929	1.568
E	0.054	0.020	0.102	0.877	0.585	1.178	0.108	0.04	0.182	1.039	0.736	1.371
O	0.014	-0.027	0.059	1.030	0.715	1.348	-0.020	-0.060	0.015	1.000	0.679	1.327
A	0.011	-0.032	0.056	1.170	0.860	1.492	-0.159	-0.224	-0.108	1.022	0.707	1.356
C	0.034	-0.006	0.0803	0.837	0.537	1.148	0.168	0.114	0.237	1.039	0.729	1.362

*Note*. M1, mediator 1 (personality); M2, mediator 2 (stress); CI, confidence interval

^#^ a1×b1, the indirect effect of gender on depressive symptoms via each personality factor

^$^ a2×b2, the indirect effect of gender on depressive symptoms via stress

^&^ the indirect effect via M1&M2, the indirect effect of gender on depressive symptoms via each personality factor and stress.

The indirect effect of gender on depression through the pathway from extraversion and stress was significant (M1&M2, coefficient: 0.108, 95% CI: 0.040, 0.182), but effect size was very small (Effect size_cs_<0.01). The indirect effects of gender on depression were significant in each pathway via extraversion (a1×b1, coefficient: 0.054, 95% CI: 0.020, 0.102) and via stress (a2×b2, coefficient: 0.877, 95% CI: 0.585, 1.178).

The indirect effect of gender on depression was significant through the pathways from agreeableness to stress (M1&M2, coefficient: -0.159; 95% CI:-0.224, -0.108) and from conscientiousness to stress (coefficient: 0.168; 95% CI: 0.114, 0.237), and their effect sizes were significant but small (>0.01). The indirect effect of gender on depression via openness and stress was not significant (M1&M2, coefficient: -0.020, 95% CI: -0.060, 0.015). There were no significant indirect effects of gender via personality factor of openness (a1×b1, coefficient: 0.014, 95% CI: -0.027, 0.059), agreeableness (coefficient: 0.011, 95% CI: -0.032, 0.056), or conscientiousness (coefficient: 0.034, 95% CI: -0.006, 0.080) after controlling for stress. Effect sizes_cs_ for the significant mediation model were ranged 0.01 to 0.05 for neuroticism, agreeableness and conscientiousness which are suggested to be small (S3 Table). The mediation models without covariates showed similar findings (S4 Table).

Thus, the pathway from gender and depressive symptoms was mediated by neuroticism and extraversion with or without a path through perceived stress. Agreeableness and conscientiousness mediated the association between gender and depression only by an indirect path through perceived stress.

### Supplementary Analysis

Because five factor personality traits were correlated among each other, it was difficult to determine unique effects of each personality trait when each personality factor was separately examined. We re-ran analyses to test unique effects of each personality trait with controlling for other traits by including all five traits simultaneously in the mediation models. The mediation model 1 showed consistent findings in neuroticism and extraversion for men and women (S5 Table). The mediation model 2 confirmed that neuroticism was a significant mediator between gender and depressive symptoms (S6 Table).

## Discussion

We found that neuroticism and extraversion were directly and indirectly associated with depressive symptoms via perceived stress, while agreeableness and conscientiousness were associated with depressive symptoms only through perceived stress. Moreover, female gender was associated with higher neuroticism and agreeableness and lower extraversion and conscientiousness, which were related to higher level of stress, and this could in turn lead to greater depressive symptoms. These findings may contribute to the understanding of how personality factors and perceived stress are related with depressive symptoms and how these factors contribute to the relationship between gender and depression. To our knowledge, this is the first study to examine the mediational roles of the five factor personality traits in relation to perceived stress on gender differences in depressive symptoms.

Personality factors are known to be involved in the regulation of emotion and cognitive vulnerability that contribute to predisposition to depression [39]. Our findings show that high neuroticism and low extraversion had direct effects on depressive symptoms and also affected the stress level, which was correlated with increased depressive symptoms. Conversely, agreeableness and conscientiousness had indirect effects on depressive symptoms, but only when mediated by stress. Individuals with high neuroticism tended to promote maladaptive cognitive processes such as ruminative thinking and hopelessness, which increased the risk of depressive symptoms [40]. Higher extraversion was associated with positive emotionality and sociability, reducing stress-related responses and depressive symptoms [5]. Agreeableness and conscientiousness have been correlated with avoiding interpersonal problems and having positive emotional coping in response to stressful situations [1, 4]. Our study supports the premise that certain types of personality factors (e.g., higher neuroticism and lower extraversion) may promote negative emotional regulation or maladaptive reactivity to stress, which increases the predisposition to depressive symptoms.

The current results indicate that female gender was associated with higher neuroticism and lower extraversion, which contributed to greater depressive symptoms directly or indirectly when mediated by stress. This result is consistent with previous findings that women scored consistently higher in neuroticism than did men across varying age ranges and world regions and that a high degree of neuroticism in adolescent girls was associated with later negative life experiences [25]. In addition, higher extraversion was associated with a reduced risk of anxiety and depressive disorders [5], while extraversion was found to have a protective effect against the risk of suicide in men [41]. Because gender differences in extraversion were not consistent with previous findings [42], it is possible that extraversion might not always explain the lower risk of depression in men.

Agreeableness and conscientiousness were previously found to be negatively associated with depressive symptoms [43], and our results demonstrated that these associations were mediated by stress in both genders. Agreeableness and conscientiousness did not solely explain the relationship between gender and depression after controlling for stress. These findings support the hypothesis that conscientiousness might play a role in stress-related vulnerability [44], which in turn might result in differential effects of gender on depression rather than as an single mediator.

It was previously reported that agreeableness has positive effects on coping with stress related with interpersonal aspects [45], but the current findings show that female gender was associated with higher agreeableness as well as greater stress and depressive symptoms. It was reported that the association between agreeableness and coping with stress was dependent on stressor types, and higher agreeableness might be less effective to cope with certain types of interpersonal stress [11]. In the present study, agreeableness showed significant but weak negative correlations with perceived stress and depressive symptoms, suggesting that higher agreeableness in women might have small effects on reducing general stress levels and depressive symptoms.

Despite the findings of significant gender differences in openness, this personality trait did not have a direct effect on depression or an indirect effect when mediated by stress. A previous study showed that higher scores in the subsets of openness to experiences was related to depression [46], while another reported that depressive individuals showed lower levels of openness to experiences [47]. In our study, openness to experience was not significantly associated with depressive symptoms in either women or men. Furthermore, gender differences in openness to experience were not associated with either higher perceived stress or greater depressive symptoms in women.

Although personality traits have been known to be important risk factors for depressive symptoms, the current study showed that the correlations between personality traits and stress or depressive symptoms had small to moderate effect sizes (r<0.4). Specifically, the correlation coefficients of agreeableness and conscientiousness with depressive symptoms or stress did not reach the minimum effect size of practical significance suggested by Ferguson [48]. One possible reason for the small effect size might be the multiple influences on depressive symptoms such as biological and environmental factors [49]. It was previously reported that several mediators account for the relationship between gender and depression [26]. However, perceived stress was only considered as a mediator with the personality factors in our analysis. So, the reasons that certain personality factors showed weak correlations with depressive symptoms might be due to various pathways including psychosocial and environmental factors by which certain personality factors could indirectly affect depression symptoms.

There are several strengths in the present study, including study sample size and methodological approaches. Although gender differences in depressive symptoms were previously reported across different countries [50], our study is first performed in the Korean population to examine the mediating roles of five factor personality traits on the link between gender and depressive symptoms. The large sample size derived from a population cohort was useful for studying the risk factors and etiology that may contribute to the development of depressive disorders in Korean adults. Despite these strengths, there are also several limitations. It was not possible to find a causal relationships among personality, perceived stress level, and depressive symptoms because one of the assumptions of mediation analysis is temporal precedence between variables [33]. Although this was a cross-sectional analysis and can therefore only be correlative, several studies support the hypothesis that personality traits as heritable and relatively stable individual characteristics are longitudinally associated with psychological distress and depressive symptoms.

In conclusion, our findings suggest that personality traits may be related with depressive symptoms through direct or indirect paths via their influence on perceived stress. Our results also showed that personality and stress play mediating roles in the link between gender and depression. Our approaches may be useful for understanding the effects of personality factors on vulnerability to stress and gender differences in depression susceptibility.

## Supporting Information

S1 Data The data underlying the findings in this study. (XLSX)

S1 Table Completely standardized effect sizes for the mediation effect of stress in the associations between personality and depression. (DOCX)

S2 Table Mediation effects of stress in the associations between personality and depression without covariates. (DOCX)

S3 Table Completely standardized effect sizes for multiple mediation effects through personality and stress in the association between gender and depression. (DOCX)

S4 Table Multiple mediation effects through personality and stress in the association between gender and depression without covariates. (DOCX)

S5 Table Mediation effects of stress in the associations between personality and depression with controlling for other personality factors. (DOCX)

S6 Table Multiple mediation effects through personality and stress in the association between gender and depression with controlling for other personality factors. (DOCX)
