# Correction: Direct and Indirect Effects of Five Factor Personality and Gender on Depressive Symptoms Mediated by Perceived Stress

**DOI:** 10.1371/journal.pone.0157204

**Published:** 2016-06-03

**Authors:** Song E. Kim, Han-Na Kim, Juhee Cho, Min-Jung Kwon, Yoosoo Chang, Seungho Ryu, Hocheol Shin, Hyung-Lae Kim

The following information is missing from the Funding section: This research was supported by the National Research Foundation of Korea, funded by the Ministry of Education (NRF-2013R1A1A2062702; http://www.nrf.re.kr/nrf_eng_cms/) and Ministry of Science, ICT & Future Planning (NRF-2014R1A2A2A04006291; http://english.msip.go.kr/english/main/main.do).
