# Correction: Correction: Direct and Indirect Effects of Five Factor Personality and Gender on Depressive Symptoms Mediated by Perceived Stress

**DOI:** 10.1371/journal.pone.0191142

**Published:** 2018-01-05

**Authors:** The PLOS ONE Staff

## Notice of Republication

This article was republished on October 3, 2016, to correct for errors in the corrected Funding section. The publisher apologizes for the errors. Please download this article again to view the correct version. The originally published, uncorrected article and the republished, corrected articles are provided here for reference.

## Supporting information

S1 File Originally published, uncorrected article. (PDF)

S2 File Republished, corrected article. (PDF)
