# Supplementary material for: Direct and Indirect Effects of Five Factor Personality and Gender on Depressive Symptoms Mediated by Perceived Stress
Source: PLoS One. 2016 Apr 27;11(4):e0154140. doi: 10.1371/journal.pone.0154140 (PMC4847785; doi:10.1371/journal.pone.0154140)
Supplement: S1 Table — (DOCX) [file pone.0154140.s002.docx]

**S1 Table. Completely standardized effect sizes for the mediation effect of stress in the associations between personality and depression**

| Men | | | |
| --- | --- | --- | --- |
| IV | Effect Size | CI lower | CI upper |
| N | 0.233 | 0.194 | 0.270 |
| E | -0.124 | -0.157 | -0.093 |
| O | -0.038 | -0.077 | -0.004 |
| A | -0.076 | -0.111 | -0.043 |
| C | -0.112 | -0.145 | -0.079 |
| Women | | | |
| IV | Effect Size | CI lower | CI upper |
| N | 0.285 | 0.261 | 0.310 |
| E | -0.147 | -0.168 | -0.123 |
| O | -0.007 | -0.030 | 0.020 |
| A | -0.097 | -0.123 | -0.071 |
| C | -0.096 | -0.122 | -0.068 |

*Note.* N, neuroticism; E, extraversion; O, openness to experience; A, agreeableness; C, conscientiousness; IV, independent variable; CI, 95% confidence interval
