# Supplementary material for: Direct and Indirect Effects of Five Factor Personality and Gender on Depressive Symptoms Mediated by Perceived Stress
Source: PLoS One. 2016 Apr 27;11(4):e0154140. doi: 10.1371/journal.pone.0154140 (PMC4847785; doi:10.1371/journal.pone.0154140)
Supplement: S2 Table — (DOCX) [file pone.0154140.s003.docx]

**S2 Table. Mediation effects of stress in the associations between personality and depression without covariates**

|  |  | **Effect of IV on M** | | **Effect of M on DV** | | **Total effect** | | **Direct effect** | | **Indirect effect** | | |
| --- | --- | --- | --- | --- | --- | --- | --- | --- | --- | --- | --- | --- |
|  | IV | a | SE | b | SE | c | SE | c' | SE | a×b | CI lower | CI upper |
| Men | N | 0.327*** | 0.019 | 0.432*** | 0.024 | 0.216*** | 0.017 | 0.075*** | 0.017 | 0.141^†^ | 0.115 | 0.169 |
|  | E | -0.144*** | 0.019 | 0.458*** | 0.022 | -0.133*** | 0.016 | -0.067*** | 0.014 | -0.066^†^ | -0.086 | -0.047 |
|  | O | -0.038* | 0.019 | 0.482*** | 0.021 | -0.018 | 0.016 | 0.000 | 0.013 | -0.018 | -0.039 | 0.002 |
|  | A | -0.090*** | 0.017 | 0.477*** | 0.021 | -0.060*** | 0.015 | -0.017 | 0.012 | -0.043^†^ | -0.061 | -0.027 |
|  | C | -0.143*** | 0.019 | 0.467*** | 0.022 | -0.110*** | 0.017 | -0.043** | 0.014 | -0.067^†^ | -0.087 | -0.048 |
| Women | N | 0.365*** | 0.013 | 0.603*** | 0.016 | 0.296*** | 0.013 | 0.076*** | 0.012 | 0.220^†^ | 0.197 | 0.241 |
|  | E | -0.154*** | 0.013 | 0.638*** | 0.014 | -0.139*** | 0.013 | -0.041*** | 0.010 | -0.098^†^ | -0.116 | -0.080 |
|  | O | 0.012 | 0.011 | 0.649*** | 0.014 | 0.022* | 0.011 | 0.014 | 0.008 | 0.008 | -0.007 | 0.022 |
|  | A | -0.109*** | 0.012 | 0.651*** | 0.014 | -0.067*** | 0.012 | 0.004 | 0.009 | -0.071^†^ | -0.086 | -0.055 |
|  | C | -0.125*** | 0.014 | 0.647*** | 0.014 | -0.092*** | 0.014 | -0.011 | 0.011 | -0.081^†^ | -0.103 | -0.062 |

*Note.* N, neuroticism; E, extraversion; O, openness to experience; A, agreeableness; C, conscientiousness; IV, independent variable; DV, dependent variable; M, mediator; SE, standard error; CI, 95% confidence interval

^*^*p*<0.05, ^**^*p*<0.01, ^***^*p*<0.001

^†^significant indirect effect
