# Supplementary material for: Direct and Indirect Effects of Five Factor Personality and Gender on Depressive Symptoms Mediated by Perceived Stress
Source: PLoS One. 2016 Apr 27;11(4):e0154140. doi: 10.1371/journal.pone.0154140 (PMC4847785; doi:10.1371/journal.pone.0154140)
Supplement: S3 Table — (DOCX) [file pone.0154140.s004.docx]

**S3 Table. Completely standardized effect sizes for multiple mediation effects through personality and stress in the association between gender and depression**

|  | **Indirect effect via M1 (a1×b1)** | | | **Indirect effect via M2 (a2×b2)** | | | **Indirect effect via M1 & M2** | | | **Indirect effect (total)** | | |
| --- | --- | --- | --- | --- | --- | --- | --- | --- | --- | --- | --- | --- |
|  | Coefficient | CI lower | CI upper | Coefficient | CI lower | CI upper | Coefficient | CI lower | CI upper | Coefficient | CI lower | CI upper |
| N | 0.020 | 0.014 | 0.026 | 0.007 | -0.009 | 0.022 | 0.052 | 0.043 | 0.062 | 0.078 | 0.059 | 0.098 |
| E | 0.003 | 0.001 | 0.006 | 0.055 | 0.037 | 0.073 | 0.007 | 0.003 | 0.011 | 0.065 | 0.046 | 0.085 |
| O | 0.001 | -0.002 | 0.004 | 0.065 | 0.045 | 0.084 | -0.001 | -0.004 | 0.001 | 0.064 | 0.044 | 0.083 |
| A | 0.001 | -0.002 | 0.004 | 0.073 | 0.055 | 0.093 | -0.010 | -0.014 | -0.007 | 0.064 | 0.045 | 0.084 |
| C | 0.002 | 0.000 | 0.005 | 0.053 | 0.034 | 0.071 | 0.011 | 0.007 | 0.015 | 0.065 | 0.046 | 0.084 |

*Note.* N, neuroticism; E, extraversion; O, openness to experience; A, agreeableness; C, conscientiousness; M1, mediator 1; M2, mediator 2; CI, 95% confidence interval
