# Supplementary material for: Direct and Indirect Effects of Five Factor Personality and Gender on Depressive Symptoms Mediated by Perceived Stress
Source: PLoS One. 2016 Apr 27;11(4):e0154140. doi: 10.1371/journal.pone.0154140 (PMC4847785; doi:10.1371/journal.pone.0154140)
Supplement: S4 Table — (DOCX) [file pone.0154140.s005.docx]

**S4 Table. Multiple mediation effects through personality and stress in the association between gender and depression without covariates**

|  | **Indirect effect via M1 (a1×b1)** | | | **Indirect effect via M2 (a2×b2)** | | | **Indirect effect via M1 & M2** | | | **Indirect effect (total)** | | |
| --- | --- | --- | --- | --- | --- | --- | --- | --- | --- | --- | --- | --- |
|  | Coefficient | CI lower | CI upper | Coefficient | CI lower | CI upper | Coefficient | CI lower | CI upper | Coefficient | CI lower | CI upper |
| N | 0.395 | 0.285 | 0.514 | 0.068 | -0.164 | 0.292 | 1.061 | 0.915 | 1.230 | 1.524 | 1.226 | 1.829 |
| E | 0.031 | 0.004 | 0.072 | 1.133 | 0.873 | 1.404 | 0.060 | 0.004 | 0.124 | 1.224 | 0.948 | 1.515 |
| O | 0.051 | -0.004 | 0.112 | 1.217 | 0.946 | 1.501 | 0.005 | -0.046 | 0.053 | 1.273 | 0.996 | 1.553 |
| A | -0.002 | -0.028 | 0.020 | 1.321 | 1.048 | 1.610 | -0.099 | -0.153 | -0.052 | 1.220 | 0.943 | 1.510 |
| C | 0.074 | 0.003 | 0.147 | 0.888 | 0.625 | 1.166 | 0.325 | 0.253 | 0.413 | 1.287 | 1.008 | 1.583 |

*Note.* N, neuroticism; E, extraversion; O, openness to experience; A, agreeableness; C, conscientiousness; M1, mediator 1; M2, mediator 2; CI, 95% confidence interval
