# Supplementary material for: Direct and Indirect Effects of Five Factor Personality and Gender on Depressive Symptoms Mediated by Perceived Stress
Source: PLoS One. 2016 Apr 27;11(4):e0154140. doi: 10.1371/journal.pone.0154140 (PMC4847785; doi:10.1371/journal.pone.0154140)
Supplement: S5 Table — (DOCX) [file pone.0154140.s006.docx]

**S5 Table. Mediation effects of stress in the associations between personality and depression with controlling for other personality factors**

|  |  | **Effect of IV on M** | | **Effect of M on DV** | | **Total effect** | | **Direct effect** | | **Indirect effect** | | |
| --- | --- | --- | --- | --- | --- | --- | --- | --- | --- | --- | --- | --- |
|  | IV | a | SE | b | SE | c | SE | c' | SE | a×b | CI lower | CI upper |
| Men | N | 2.934*** | 0.182 | 0.423*** | 0.023 | 1.838*** | 0.161 | 0.597*** | 0.157 | 1.242^†^ | 0.994 | 1.509 |
|  | E | -0.752*** | 0.174 | 0.423*** | 0.023 | -0.977*** | 0.154 | -0.659*** | 0.136 | -0.318^†^ | -0.485 | -0.179 |
|  | O | 0.190 | 0.198 | 0.423*** | 0.023 | 0.168 | 0.175 | 0.087 | 0.154 | 0.081 | -0.102 | 0.259 |
|  | A | -0.343 | 0.176 | 0.423*** | 0.023 | -0.105 | 0.155 | 0.040 | 0.137 | -0.145 | -0.297 | 0.007 |
|  | C | -0.360* | 0.174 | 0.423*** | 0.023 | -0.215 | 0.154 | -0.062 | 0.135 | -0.153 | -0.310 | 0.002 |
| Women | N | 0.349*** | 0.014 | 0.591*** | 0.016 | 0.289*** | 0.015 | 0.082*** | 0.013 | 0.207^†^ | 0.183 | 0.233 |
|  | E | -0.095*** | 0.014 | 0.591*** | 0.016 | -0.095*** | 0.014 | -0.042*** | 0.011 | -0.056^†^ | -0.073 | -0.039 |
|  | O | 0.029** | 0.011 | 0.591*** | 0.016 | 0.038** | 0.011 | 0.021* | 0.009 | 0.017^†^ | 0.004 | 0.030 |
|  | A | -0.032** | 0.011 | 0.591*** | 0.016 | -0.003 | 0.011 | 0.016 | 0.009 | -0.019^†^ | -0.033 | -0.006 |
|  | C | 0.060*** | 0.014 | 0.591*** | 0.016 | 0.061*** | 0.015 | 0.025* | 0.012 | 0.036^†^ | 0.017 | 0.054 |

*Note.* N, neuroticism; E, extraversion; O, openness to experience; A, agreeableness; C, conscientiousness; IV, independent variable; DV, dependent variable; M, mediator; SE, standard error; CI, 95% confidence interval

^*^*p*<0.05, ^**^*p*<0.01, ^***^*p*<0.001

^†^significant indirect effect
