# Supplementary material for: Direct and Indirect Effects of Five Factor Personality and Gender on Depressive Symptoms Mediated by Perceived Stress
Source: PLoS One. 2016 Apr 27;11(4):e0154140. doi: 10.1371/journal.pone.0154140 (PMC4847785; doi:10.1371/journal.pone.0154140)
Supplement: S6 Table — (DOCX) [file pone.0154140.s007.docx]

**S6 Table. Multiple mediation effects through personality and stress in the association between gender and depression with controlling for other personality factors**

|  | **Indirect effect via M1 (a1×b1)** | | | **Indirect effect via M2 (a2×b2)** | | | **Indirect effect via M1 & M2** | | | **Indirect effect (total)** | | |
| --- | --- | --- | --- | --- | --- | --- | --- | --- | --- | --- | --- | --- |
|  | Coefficient | CI lower | CI upper | Coefficient | CI lower | CI upper | Coefficient | CI lower | CI upper | Coefficient | CI lower | CI upper |
| N | 0.248 | 0.169 | 0.347 | 0.139 | -0.118 | 0.390 | 0.620 | 0.495 | 0.754 | 1.007 | 0.696 | 1.316 |
| E | 0.032 | 0.003 | 0.071 | 0.139 | -0.112 | 0.402 | 0.029 | 0.002 | 0.062 | 0.200 | -0.052 | 0.471 |
| O | 0.061 | 0.016 | 0.115 | 0.139 | -0.111 | 0.399 | 0.044 | 0.013 | 0.079 | 0.245 | -0.012 | 0.503 |
| A | 0.057 | 0.000 | 0.117 | 0.139 | -0.107 | 0.398 | -0.053 | -0.100 | -0.014 | 0.143 | -0.105 | 0.399 |
| C | -0.017 | -0.047 | -0.002 | 0.139 | -0.112 | 0.397 | -0.025 | -0.054 | -0.005 | 0.097 | -0.157 | 0.360 |

*Note.* N, neuroticism; E, extraversion; O, openness to experience; A, agreeableness; C, conscientiousness; M1, mediator 1; M2, mediator 2; CI, 95% confidence interval
