# Supplementary material for: Correction: Correction: Direct and Indirect Effects of Five Factor Personality and Gender on Depressive Symptoms Mediated by Perceived Stress
Source: PLoS One. 2018 Jan 5;13(1):e0191142. doi: 10.1371/journal.pone.0191142 (PMC5755933; doi:10.1371/journal.pone.0191142)
Supplement: S1 File — (PDF) [file pone.0191142.s001.pdf]

PLOS | ONE

CORRECTION

Correction: Direct and Indirect Effects of Five Factor Personality and Gender on Depressive Symptoms Mediated by Perceived Stress

Song E. Kim, Han-Na Kim, Juhee Cho, Min-Jung Kwon, Yoosoo Chang, Seungho Ryu, Hocheol Shin, Hyung-Lae Kim

The following information is missing from the Funding section: This research was supported by the National Research Foundation of Korea, funded by the Ministry of Education (http://www.nrf.re.kr/nrf_eng_cms/) and Ministry of Science, ICT & Future Planning (http://english.msip.go.kr/english/main/main.do).

Reference

1. Kim SE, Kim H-N, Cho J, Kwon M-J, Chang Y, Ryu S, et al. (2016) Direct and Indirect Effects of Five Factor Personality and Gender on Depressive Symptoms Mediated by Perceived Stress. PLoS ONE 11(4): e0154140. doi: 10.1371/journal.pone.0154140 PMID: 27120051

CrossMark
click for updates

OPEN ACCESS

Citation: Kim SE, Kim H-N, Cho J, Kwon M-J, Chang Y, Ryu S, et al. (2016) Correction: Direct and Indirect Effects of Five Factor Personality and Gender on Depressive Symptoms Mediated by Perceived Stress. PLoS ONE 11(6): e0157204. doi:10.1371/journal.pone.0157204

Published: June 3, 2016

Copyright: © 2016 Kim et al. This is an open access article distributed under the terms of the Creative Commons Attribution License, which permits unrestricted use, distribution, and reproduction in any medium, provided the original author and source are credited.
